# Cellular Adhesion Promotes Prostate Cancer Cells Escape from Dormancy

**DOI:** 10.1371/journal.pone.0130565

**Published:** 2015-06-19

**Authors:** Nazanin Ruppender, Sandy Larson, Bryce Lakely, Lori Kollath, Lisha Brown, Ilsa Coleman, Roger Coleman, Holly Nguyen, Peter S. Nelson, Eva Corey, Linda A. Snyder, Robert L. Vessella, Colm Morrissey, Hung-Ming Lam

**Affiliations:** 1 Department of Urology, University of Washington, Seattle, Washington, United States of America; 2 Divison of Human Biology, Fred Hutchinson Cancer Research Center, Seattle, Washington, United States of America; 3 Department of Medicine, University of Washington, Seattle, Washington, United States of America; 4 Janssen Research and Development, LLC, Spring House, Pennsylvania, United States of America; 5 Department of Veterans Affairs Medical Center, Seattle, Washington, United States of America; Thomas Jefferson University, UNITED STATES

## Abstract

Dissemination of prostate cancer (PCa) cells to the bone marrow is an early event in the disease process. In some patients, disseminated tumor cells (DTC) proliferate to form active metastases after a prolonged period of undetectable disease known as tumor dormancy. Identifying mechanisms of PCa dormancy and reactivation remain a challenge partly due to the lack of *in vitro* models. Here, we characterized *in vitro* PCa dormancy-reactivation by inducing cells from three patient-derived xenograft (PDX) lines to proliferate through tumor cell contact with each other and with bone marrow stroma. Proliferating PCa cells demonstrated tumor cell-cell contact and integrin clustering by immunofluorescence. Global gene expression analyses on proliferating cells cultured on bone marrow stroma revealed a downregulation of TGFB2 in all of the three proliferating PCa PDX lines when compared to their non-proliferating counterparts. Furthermore, constitutive activation of myosin light chain kinase (MLCK), a downstream effector of integrin-beta1 and TGF-beta2, in non-proliferating cells promoted cell proliferation. This cell proliferation was associated with an upregulation of CDK6 and a downregulation of E2F4. Taken together, our data provide the first clinically relevant *in vitro* model to support cellular adhesion and downregulation of TGFB2 as a potential mechanism by which PCa cells may escape from dormancy. Targeting the TGF-beta2-associated mechanism could provide novel opportunities to prevent lethal PCa metastasis.

## Introduction

The dissemination of prostate cancer (PCa) cells to the bone marrow is an early event in the PCa disease process [1, 2]. In many cases, these disseminated tumor cells (DTC) proliferate to form active metastases after a prolonged period of undetectable disease following prostatectomy. This latency period is often referred to as tumor dormancy. To date, dormancy remains a significant clinical challenge, as PCa patients presented with bone metastases ultimately stop responding to second line therapies and eventually succumb to the disease. Thus, it has become paramount to identify mechanisms of tumor dormancy in an effort to prevent PCa recurrence.

A dormant tumor cell does not actively proliferate, yet has the potential to multiply given the right external cues. By this definition, multiple scenarios could potentially induce dormancy, including unfavorable tumor microenvironment, nutrient starvation, the inherent nature of the DTC, or epigenetic changes caused by the microenvironment [2, 3]. However, not all instances of indolent PCa necessarily constitute dormancy. A patient may simply have slow-growing tumor cells residing at the metastatic site at the time of initial treatment and experience recurrence shortly thereafter. Others may never experience recurrence, while a subset of patients experience recurrence only after extended periods. To date, the mechanisms of dormancy remain largely unknown. However, the urokinase-like plasminogen activator (uPA) and its associated receptor (uPAR) have been implicated in the regulation of dormancy in various cancers. Specifically, high levels of uPA and uPAR induce dormancy escape by upregulating ERK/p38 ratio within cancer cells [4, 5]. This high uPAR expression was associated with the activation of α_v_β_1_ integrin, resulting in tumor growth [4–7]. In a separate study, uPA-regulated migration of tumor cells was activated by the myosin light chain kinase (MLCK) [8] which phosphorylation was induced by ERK [9]. MLCK is a known regulator of contractility, motility, and adhesion [10, 11], however the role of MLCK in PCa dormancy escape remains unknown.

Matrix and intercellular adhesions has been implicated in tumor dormancy regulation. Studies showed that integrin-mediated cellular adhesion to the extracellular matrix activates MAPKs [12–14] which regulates tumor growth [15–19]. In PCa, upregulation of β_1_ integrin promotes the growth and invasion of cells [3, 20], and interactions between tumor and stroma may be attributable to the escape of dormant cells from radiotherapy [21]. Recent studies examining human PCa cell lines on mouse bone marrow stroma have identified important factors in the mouse hematopoietic niche that regulate dormancy [22, 23].

We here characterized the dormancy and growth of three PCa patient-derived xenografts (PDXs) established from metastases obtained at rapid autopsy or surgery on human bone marrow microenvironment *in vitro*. These PDXs (LuCaP 86.2, 92, and 93) displayed *in vitro* quiescence in typical cell culture conditions which may represent dormancy and we aimed to identify the role of cell-cell adhesion in the release of PCa from dormancy in a human bone marrow context. We determined that tumor cell-cell contact on bone marrow stroma is necessary for LuCaP PDX cells to proliferate *in vitro* and was associated with a universal downregulation of TGFB2. Furthermore, LuCaP PDXs dormancy reactivation could be recapitulated by constitutively activating MLCK and cyclin-dependent kinase 6 (CDK6).

## Materials and Methods

### Dissociation, isolation, and culture of LuCaP PDX *in vitro*

Bone marrow stromal cells (BMSC) that were isolated from a patient with PCa bone metastases (BM2508) were seeded at 50,000 cells/cm^2^ overnight. The following day, BM2508 cells were treated with 10 μg/mL mitomycin C (Sigma, St Louis, MO) for 1 hour. LuCaP PDXs that were routinely passaged *in vivo* as described previously [21] were excised and dissociated using the Miltenyi human tumor dissociation kit (Miltenyi Biotec Inc., San Diego, CA; #130-95-929) and enriched by positive selection using magnetic microbeads against human epithelial antigen EpCAM/CD326 (Miltenyi Biotec Inc.; #130-061-101) according to the manufacturer’s instructions. LuCaP PDX cells were then seeded on top of the BM2508 cells at either 50,000 cells/cm^2^ (G; growing/proliferating) or 50 cells/cm^2^ (NG; not growing/dormant). At day 8, the LuCaP PDX cells were differentially trypsinized and enriched by positive and negative selection with magnetic beads, fluorescently labeled for EpCAM/CD326 and individually plucked with a micromanipulator as described previously [24]. Furthermore, to ensure that NG cells were dormant instead of senescent, a β-galactosidase assay (Pierce Biotechnology, Inc., Waltham, MA; #75707) was performed on all NG LuCaP PDX cells according to manufacturer’s instructions. All procedures involving human subjects were approved by the Institutional Review Board of the University of Washington Medical Center and all subjects signed informed consent. The animal study was specifically approved by the University of Washington Institutional Animal Care and Use Committee and all animal procedures were performed in compliance with the NIH guidelines.

### Immunofluorescent staining

The LuCaP PDX cells were dissociated, selected and plated as described above on glass coverslips conjugated with lysine. At day 8, cells were fixed with ice-cold methanol, blocked with 5% horse-goat-chicken serum and stained for EpCAM and Ki67 or _1_ integrin using a FITC-conjugated mouse monoclonal anti-human Ber-EP4 antibody (DAKO, Carpinteria, CA; F086001), and a rabbit polyclonal anti-human Ki-67 antibody (Santa Cruz Biotechnology, Dallas, TX; SC-15402) or a rabbit polyclonal anti-human ITGB1 antibody (Santa Cruz Biotechnology; SC-9970) in conjunction with a goat anti-rabbit Alexa-Fluor 546 conjugated secondary antibody (Life Technologies, Carlsbad, CA; A-11035). Coverslips were then mounted with ProLong Gold antifade reagent containing DAPI (Life Technologies; P-36931).

### Cell count and WST-1 assays

C4-2B (a gift from Dr. Leland Chung; [25]) and dissociated LuCaP PDX cells (LuCaP 86.2, 92, 93, 96, 141; [26–28]) were plated in RPMI-1640 or MEM (Life Technologies) respectively supplemented with 10% fetal bovine serum (FBS). Cells were seeded either sparsely (50 cells/cm^2^) or densely (50,000 cells/cm^2^) on a confluent monolayer of BMSC (50,000 cells/cm^2^) that was pretreated with 10 μg/ml mitomycin C. For C4-2B cells seeded sparsely on BMSC, after 1 and 8 days, cells were stained for EpCAM, Ki67, and DAPI as described above. Only EpCAM+ epithelial cells (representing C4-2B cells because BMSC are EpCAM-) were counted in the whole chamber under a fluorescent microscope using 200× magnification. For C4-2B cells seeding without BMSC, WST-1 assay (Roche Diagnostics Corporation, Indianapolis, IN) was carried out according to the manufacturer’s instructions. Absorbance was read on a microplate reader at 450 nm, and the background absorbance (media only) was subtracted from all readings. For LuCaP PDX cells, after 3, 7, and 14 days on BMSC, cells were trypsinized, stained for EpCAM and Ki67 as described above, and resuspended in 50 μl of ProLong Gold antifade reagent containing DAPI. Two-aliquots of 10 μl of stained cells were counted and EpCAM+ epithelial cells (i.e. LuCaP PDX cells) were recorded.

### Flow cytometric analysis

C4-2B cells were cultured overnight in RPMI-1640 medium supplemented with 10% FBS and then treated with DMSO or ML-7 (10μM) for 24h and 48h. The treated cells were trypsinized, fixed, stained with 10 μg/μL DAPI (4',6-diamidino-2-phenylindole, Life Technologies)/1% NP-40 /10% DMSO, and lysed using 25G syringe. At least 10,000 stained cells were analyzed using BD LSR II Flow Cytometer System (BD Biosciences, San Jose, CA) and cell cycle was analyzed by MultiCycle (De Novo Software, Glendale, CA)

### Viral transduction and drug treatments of LuCaP PDX cells *in vitro*

LuCaP PDXs were dissociated and selected as described above and plated at 50,000 cells/cm^2^ without stromal cells. The following day, cells were transduced at an MOI (multiplicity of infection) of 10 with one of the following: an adenoviral vector that either contained an activated form of myosin light chain kinase (A-tMK, a gift from Drs. Zuzana Strakova, Jody Martin, and Primal de Lanerolle**,** [29]), an empty vector, or a lentiviral vector that contained either cDNA for CDK6 or GFP (Applied Biological Materials, pLentiIII-EF1α). Cells were transduced in MEM supplemented with 10% FBS and 8 μg/mL polybrene (Santa Cruz Biotechnology). Cells were either immunofluorescently stained as described above or trypsinized for RNA extraction. To determine whether MLCK activity is necessary for PCa proliferation, C4-2B cells, which readily proliferate *in vitro*, were plated at 50,000 cells/cm^2^ in RPMI medium (Life Technologies) supplemented with 10% FBS. The cells were then either treated with 10 μM ML-7 (Sigma; I2764) or DMSO control for 24 hours.

### RNA extraction and amplification

For 10-cell transcriptomic study, 10 individually isolated cells per xenograft line were lysed and amplified cDNA was generated from the total RNA using the NuGEN Ovation RNA Amplification System as described previously [24, 30]. The cDNA was arrayed on Agilent 44K whole human genome expression oligonucleotide microarrays (Agilent Technologies, Inc.). For other *in vitro* experiments, RNA was isolated using the RNEasy mini kit (Qiagen Inc., Valencia, CA). One microgram of RNA was reverse transcribed using the Qiagen RT^2^ first strand kit, followed by PCR array or RT-qPCR analysis.

### Labeling and hybridization of amplified material to whole human genome expression oligonucleotide microarrays

Amplified cDNA from each sample was labeled using the BioPrime Total Genomic Labeling System (Life Technologies, Grand Island, NY) and microarray was performed according to previous procedures [24, 30] with slight modifications. Briefly, hybridization probes were prepared by combining 4 μg of Alexa Fluor 3-labeled sample with 400 ng Alexa Fluor 5-labeled reference. The probes were denatured at 95°C and hybridized at 63°C on Agilent Human 4x44K microarrays (Agilent Technologies, Inc., Santa Clara, CA), washed, and fluorescent array images were collected using the Agilent DNA microarray scanner. The data were loess normalized within arrays and quantile normalized between arrays in R using the Limma Bioconductor package. Data were filtered to remove probes with mean signal intensities below 300. The Statistical Analysis of Microarray (SAM) program (http://www-stat.stanford.edu/~tibs/SAM/) [31] was used to analyze expression differences between groups using unpaired, two-sample t-tests and controlled for multiple testing by estimating q-values using the false discovery rate (FDR) method. Microarray data are deposited in the Gene Expression Omnibus database under the accession number GSE64262.

### Gene expression analyses

To determine whether differential transcription observed in NG (not growing/dormant) versus G (growing/proliferating) groups were enriched for genes within canonical pathways and Gene Ontology gene sets, the t-test results were subjected to Gene Set Enrichment Analysis (GSEA) using preranked mode with permutation testing of the gene sets to adjust for multiple hypothesis testing, generating an FDR. Unsupervised, hierarchical clustering of the most differentially expressed was performed between NG and G groups based on SAM score (SAM score >2 and p≤0.05, a total of 238 genes) using Cluster 3.0 (bonsai.hgc.jp/~mdehoon/software/cluster/software.htm) and Java TreeView (http://jtreeview.sourceforge.net/).

### Ingenuity Pathway Analysis

The 238 differentially expressed genes between NG and G groups were imported into Ingenuity Pathway Analysis (IPA, Ingenuity Systems; https://www.ingenuity.com) to identify molecular and cellular functions and upstream regulators involved in cell proliferation or dormancy as previously described [30].

### Quantitative real-time PCR

For PCR array, 25ng of cDNA was used for human cell-cycle PCR array (Qiagen Inc., PAHS-020Z) according to manufacturer’s instructions. For qPCR analysis, 2ng (G and NG 10-cell study) or 10ng (lentiviral transduction studies) of cDNA was used for the Platinum SYBR Green qPCR SuperMix-UDG system (Life Technologies, 11733–038) in conjunction with the following primers: CDK6: (F) 5’AGGCTGCTGTTTTCTCTCCA3’, (R) 5’CCACACTGCTTCTTGGGTCT3’; E2F4: (F) 5’TGATGTGCCTGTTCTCAACC3’, (R) 5’GAGTCCTGTTCCCCTGCTCT3’.; RPS15: (F) 5’TCCGGCAAGATGGCAGAAGTAG3’, (R) 5’CCACGCCGCGGTAGGT3’; CDC42: (F) 5’GTCACAGTTATGATTGGTGGAGA3’, (R) 5’ TCAGCGGTCGTAATCTGTCA3’; FN1: (F) 5’AAGAGGCAGGCTCAGCAAAT3’, (R) 5’ GTCATAACAACCGGGCTTGC3’; TGFB2: (F) 5’TCTTCCCCTCCGAAAATGCC3’, (R) 5’ TCTCCATTGCTGAGACGTCAA3’.

## Results

### PDX cells require cell-cell contact to proliferate *in vitro*

**Fig 1:**
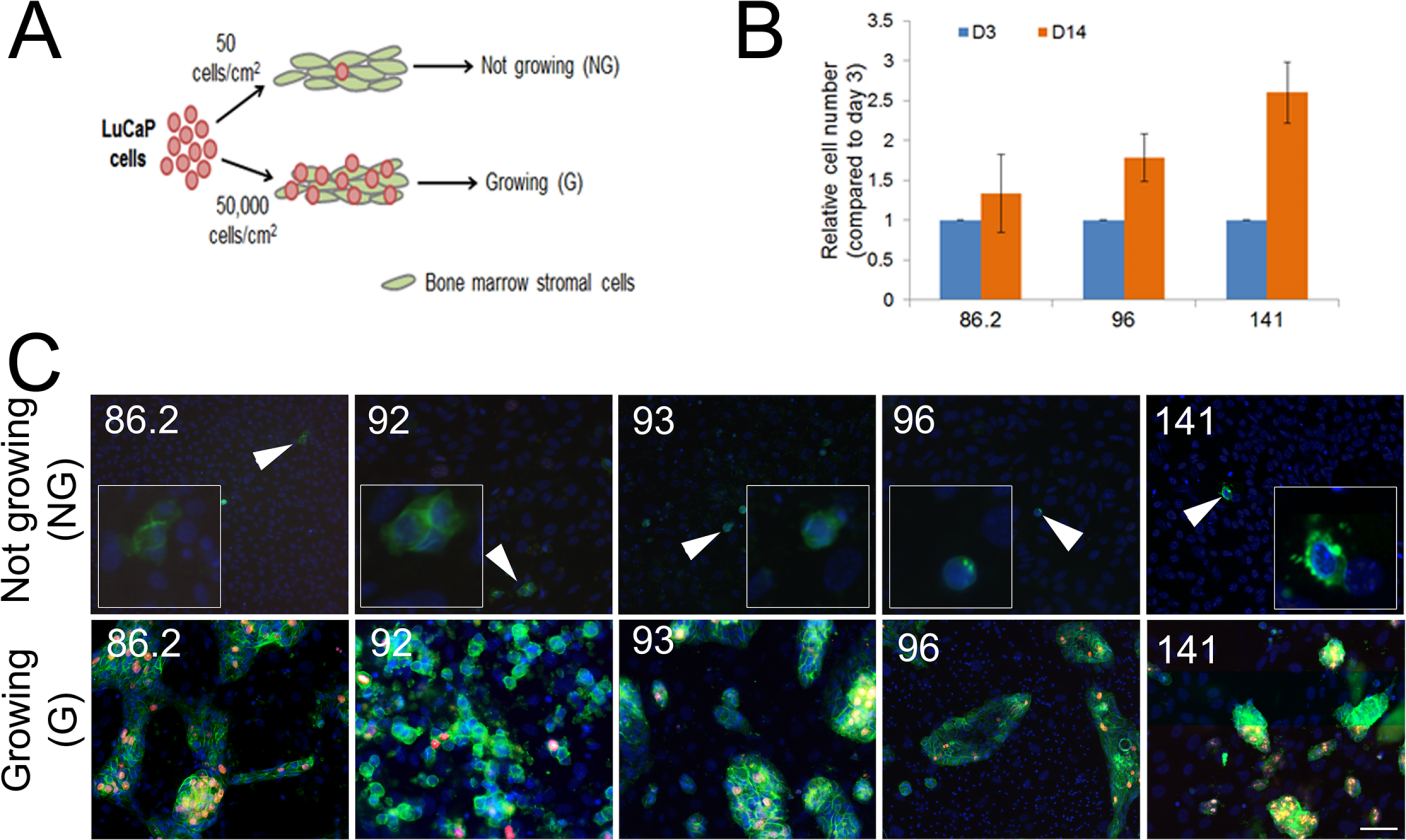
LuCaP PCa PDX cells grow on a monolayer of bone marrow stromal cells (BMSC) when seeded densely. A) A scheme showing the *in vitro* culture condition for not growing (NG) and growing (G) LuCaP PDX cells on BMSC. LuCaP cells were seeded sparsely at 50 cells/cm^2^ or densely at 50,000 cells/cm^2^ on a confluent layer of BMSC (50,000 cells/cm^2^) pretreated with 10 μg/mL mitomycin C to inhibit BMSC cell division. B) LuCaP cells seeded densely on BMSC were quantified by positive EpCAM staining on day 3, 7, and 14 post-seeding. C) LuCaP cells (86.2, 92, 93, 96, and 141) were seeded sparsely (upper panel; NG) or densely (lower panel; G) on BMSC. After 14 days, cells were fixed with ice-cold methanol and fluorescently stained with Ki67 to assess proliferation. Green, EpCAM; Red, Ki67; Blue, DAPI. White arrow: sparsely seeded cells showing negative Ki67 staining after 14 days. Magnification: 200x. Scale bar: 50 μm. Experiments were repeated 2–3 times and graphs showing mean ± SEM or representative pictures were shown.

The PCa xenografts we have established from metastases obtained at rapid autopsy or surgery do not proliferate *in vitro* after dissociation under standard monoculture conditions [32]. Of the five LuCaP PCa PDX lines we studied, none of them displayed measurable β-galactosidase activity (data not shown), suggesting these cells are dormant rather than senescent. As dormant cells by definition retain the potential to proliferate, we sought to determine whether these xenografts could be “activated” *in vitro*. We developed an *in vitro* model recapitulating the PCa cells in contact with BMSC and allowed the LuCaP PDX cells seeded either sparsely without tumor-tumor cell contact (NG, not growing, 50 cells/cm^2^) or densely where cells were in direct contact with each other (G, growing, 50,000 cells/cm^2^; Fig 1A). We here reported that when LuCaP 86.2, 96, and 141 were seeded densely on a monolayer of BMSC, they showed an increase in cell number after 14 days, whereas the cells that were seeded sparsely failed to proliferate (Fig 1B and data not shown). In contrast, C4-2B cells seeded sparsely on BMSC showed an increase in cell number after 7 days (S1 Fig). To visually detect the association between tumor cell-cell contact and proliferation, we expanded the study to five LuCaP lines for immunofluorescent detection. After 14 days in culture, no positive Ki67 staining was detected in NG cells that were sparsely seeded without tumor cell-cell contact (Fig 1C, upper panel). Consistent with the trypan blue exclusion assay, positive Ki67 staining was observed in G cells that were densely seeded and displayed cell-cell contact, suggesting that tumor cell-cell contact was associated with cell proliferation (Fig 1C, lower panel).

### β1 Integrin activity associates with LuCaP PDX cell proliferation *in vitro*

**Fig 2:**
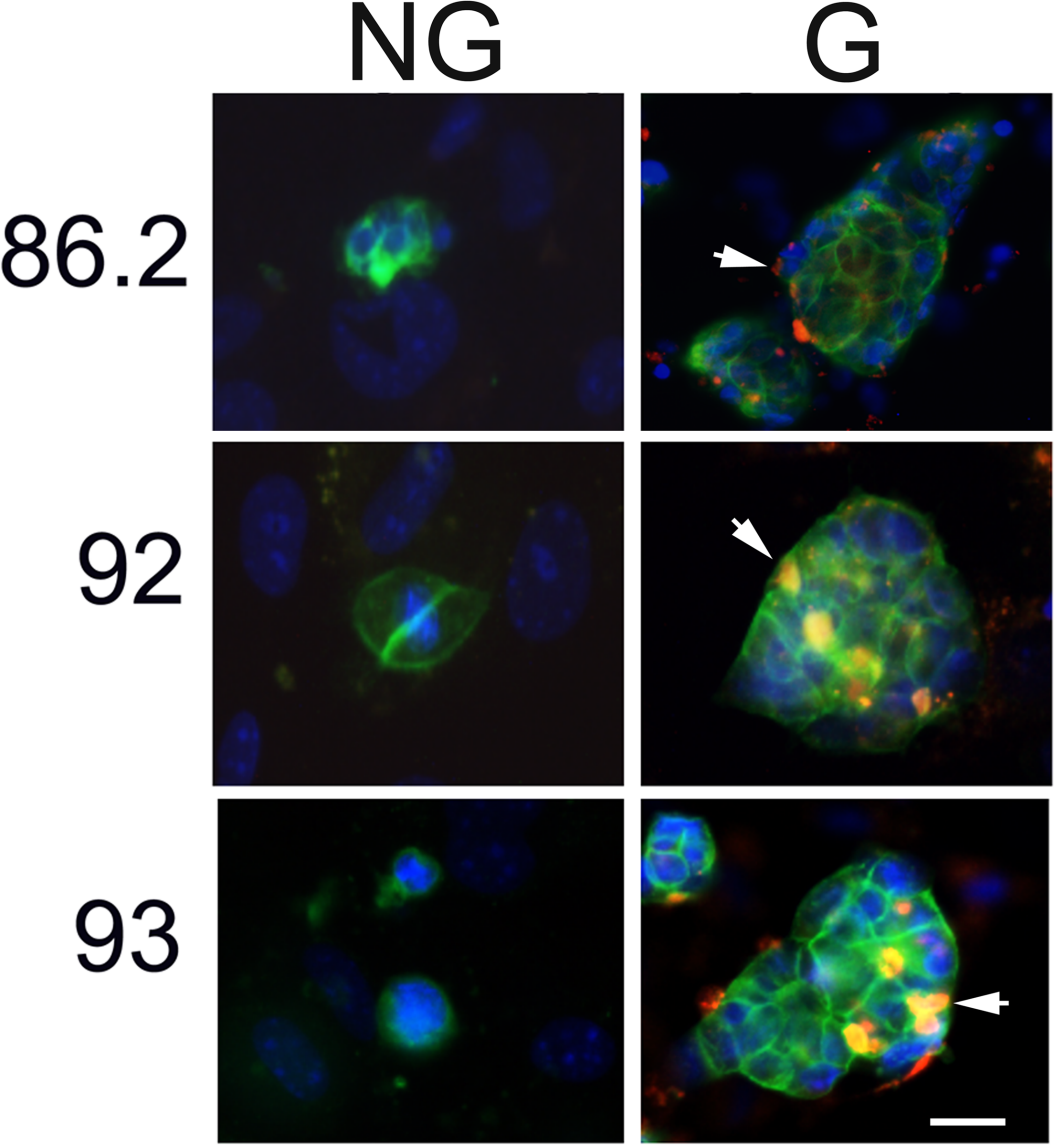
β_1_ integrin clusters were observed in LuCaP PDX cells proliferating *in vitro*. LuCaP 86.2, 92, and 93 were dissociated and cultured on a confluent monolayer of BMSC. β_**1**_ integrin immunofluorescent staining revealed integrin clustering (red clusters and highlighted by the white arrow) in densely seeded cells that were growing (G). This clustering was not detected in sparsely seeded, nonproliferative cells (not growing, NG). Green, EpCAM; Red, β1 integrin; Blue, DAPI. Magnification: 200x. Scale bar: 20 μm.

When direct cell-cell contact occurs, integrins were reported to be activated resulting in cell cycle progression and cell proliferation [33]. We therefore examined in LuCaP PDX cells whether β_1_ integrin clustering was activated in response to cell-cell contact. In LuCaP 86.2, 92, and 93, when seeded densely on a monolayer of BMSC, the proliferating G cells showed clustering of β_1_ integrin, whereas the non-proliferating NG cells seeded sparsely did not display clustering of β_1_ integrin (Fig 2).

### Gene expression analysis revealed downregulation of TGFB2 in proliferating cells

**Fig 3:**
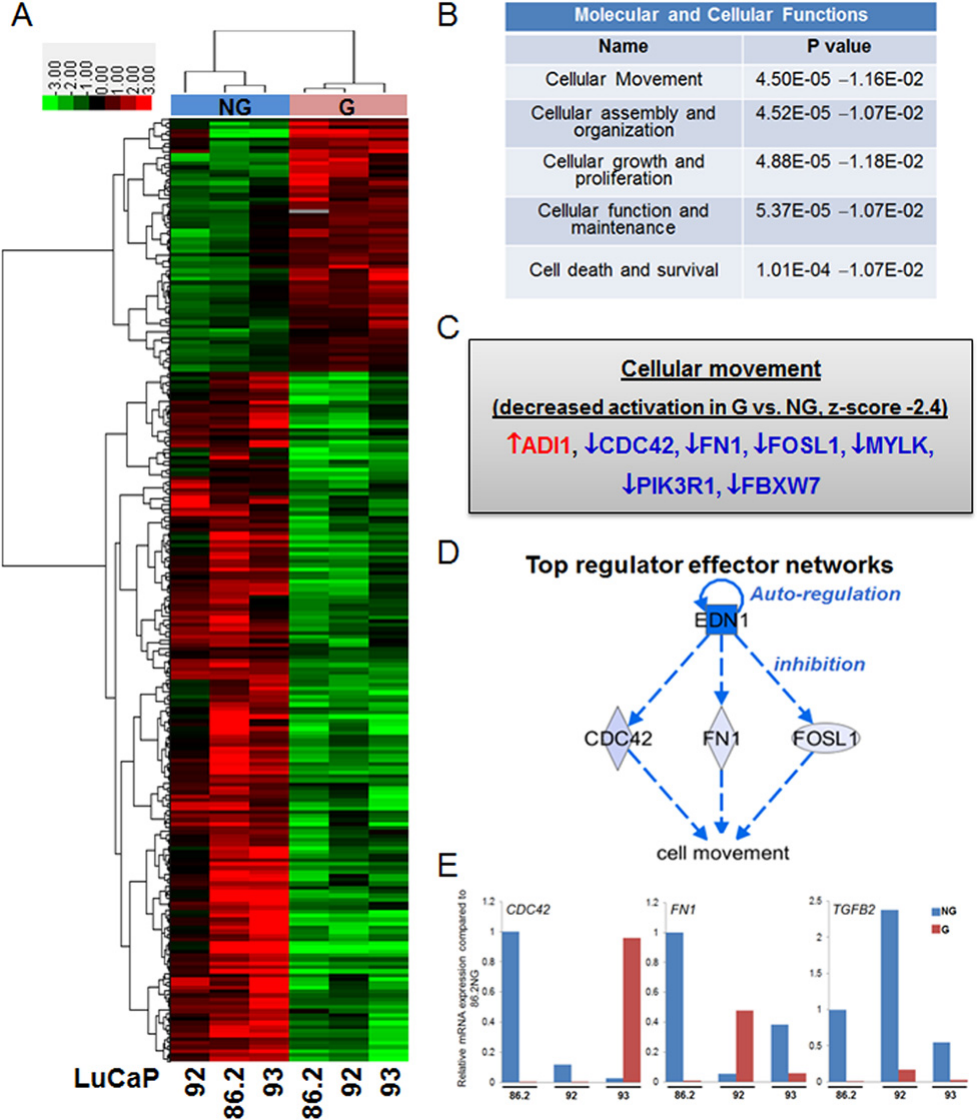
Genes associated with cellular movement were downregulated in proliferating LuCaP cells. A) Heat map of hierarchically clustered differential gene expression in NG and G LuCaP PDX cells. Green, downregulated; red, upregulated. B) Ingenuity pathway analysis showing cellular movement was the top molecular and cellular function altered between NG and G cells. C) List of eight genes that were involved in the decreased activation of cellular movement in G when compared to NG cells. D) EDN1 was predicted to be the top regulator that affected the cell movement via downregulation of FN1, CDC42, and FOSL1. E) Quantitative real-time PCR showed a downregulation of FN1, CDC42 and TGFb2 in growing LuCaP lines. Data were normalized to the levels of housekeeping gene RPS15. NG: not growing; G: growing.

Next, we conducted microarray gene expression analysis to delineate the mechanisms underlying the activation of β_1_ integrin and cell proliferation. A total of 238 genes (SAM score ≥2 or ≤-2, p<0.05) were differentially expressed between dormant/not growing (NG) and proliferating/growing (G) cells in LuCaP 86.2, 92, and 93 (Fig 3A). We observed that cellular movement was the top molecular and cellular function altered (Fig 3B, p<0.05) and a decreased activation was predicted (Fig 3C, z-score -2.4) in G when compared to NG cells. Interestingly, Ingenuity Pathway Analysis identified a top regulator effector network for those genes involved in the decreased activation on cellular movement and demonstrated that endothelin 1 (EDN1) was the common upstream regulator for the downregulation of CDC42, FN1, and FOSL1, which resulted in decreased cell movement (Fig 3C and 3D). Despite EDN1 being identified as the top common upstream regulator for decreased cellular movement in G when compared to NG cells, microarray expression analysis showed that it was not significantly altered in G when compared to NG cells (1.2 fold upregulation in G cells with a SAM score 0.2, data not shown). Since *FOSL1* has a very low endogenous level, therefore we focused on validating *CDC42* and *FN1* using real-time qPCR and found that both genes were downregulated in G cells in two of the three LuCaP PDX lines tested (Fig 3E). Furthermore, TGFβ2 is a known downstream effector of β_1_ integrin and upregulation of *TGFB2* expression has been reported to be associated with migration and cancer dormancy [34, 35], we examined and found that *TGFB2* expression was consistently downregulated in G when compared to NG cells in all three LuCaP PDX lines by real-time qPCR (Fig 3E). In clinically derived DTC isolated from the bone marrow, we validated that *FN1* (p<0.01, from gene expression dataset GSE48995, [26]) and *TGFB2* [35] were upregulated in patients with no evidence of disease when compared to patients with active PCa metastasis, whereas no significant gene expression change for *CDC42* (p = 0.67) was detected between the two groups of DTC (data not shown).

### Activation of MLCK promoted PCa PDX cells proliferation via CDK6 in the absence of BMSC

**Fig 4:**
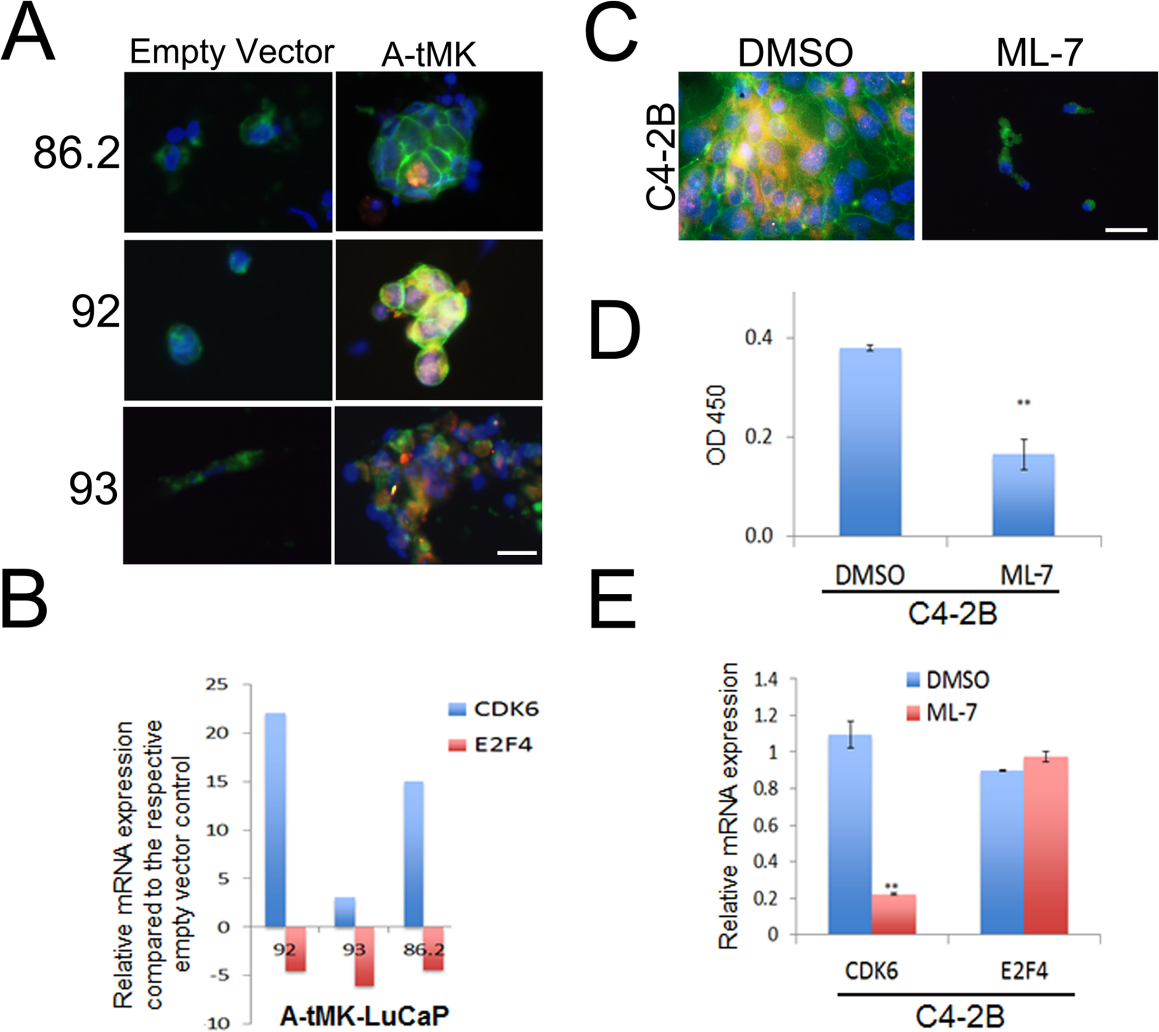
Constitutive activation of MLCK promotes proliferation of LuCaP PDX cells via upregulation of CDK6. A) LuCaP cells were infected with lentivirus containing A-tMK (constitutively activate MLCK) showed positive Ki67 staining, whereas cells transduced with an empty vector did not. B) In LuCaP 86.2, 92, and 93, ectopic expression of A-tMK induced an upregulation of CDK6 and a concurrent downregulation of E2F4 when compared to that of the empty vector-transduced cells. Inhibition of MLCK with the MLCK inhibitor ML-7 suppressed proliferation by C) abolishing Ki67 expression, D) decreasing cell viability assessed by WST-1 assay and E) downregulating CDK6 expression. E2F4 expression was not altered by the ML-7. Green, EpCAM; Red, Ki67; Blue, DAPI. Magnification: 200x. Scale bar: 20 μm. **p< 0.01 as compared to the DMSO control. CDK6: cyclin-dependent kinase 6; E2F4: E2F transcription factor 4.

Myosin light chain kinase (MLCK) is a common effector for β_1_ integrin, CDC42 and TGFβ2 and its activation has been implicated in cell proliferation [36–38]. To determine if activation of MLCK is involved in LuCaP PDX cell proliferation, we virally transduced a constitutively active form of MLCK (A-tMK) in LuCaP PDX cells. A-tMK transduction in LuCaP 86.2, 92, and 93 cells that normally do not proliferate resulted in cell clustering and positive Ki-67 expression, whereas cells transduced with an empty vector did not show cell clustering or positive Ki67 staining (Fig 4A). Gene expression analysis focusing on cell cycle regulators demonstrated that A-tMK-transduced LuCaP cells expressed an upregulated level of cyclin-dependent kinase 6 (CDK6, 3 to 22 fold) and a concurrent downregulated level of E2F transcription factor 4 (E2F4, 4 to 6 fold; Fig 4B).

To validate the involvement of MLCK activation in cell proliferation, we inhibited MLCK in C4-2B cells that readily proliferate *in vitro* in an attempt to inhibit cell proliferation. Upon MLCK inhibition by the MLCK inhibitor ML-7, C4-2B cell proliferation was reduced as evidenced by the loss of Ki67 staining (Fig 4C), the decrease in WST-1 absorbance (Fig 4D), and the arrest of cells in the G1 phase (S2 Fig). In addition, the decrease in cell proliferation was accompanied by a 4.9-fold downregulation in CDK6 mRNA expression in C4-2B cells treated with ML-7 (p = 0.007). The expression of E2F4, however, did not show a significant upregulation in C4-2B cells (Fig 4E). Collectively, the data suggested that activation of MLCK played a role in stimulating cell proliferation which is associated with an upregulation of CDK6.

### Overexpression of CDK6 facilitates the proliferation of LuCaP xenografts *in vitro*

**Fig 5:**
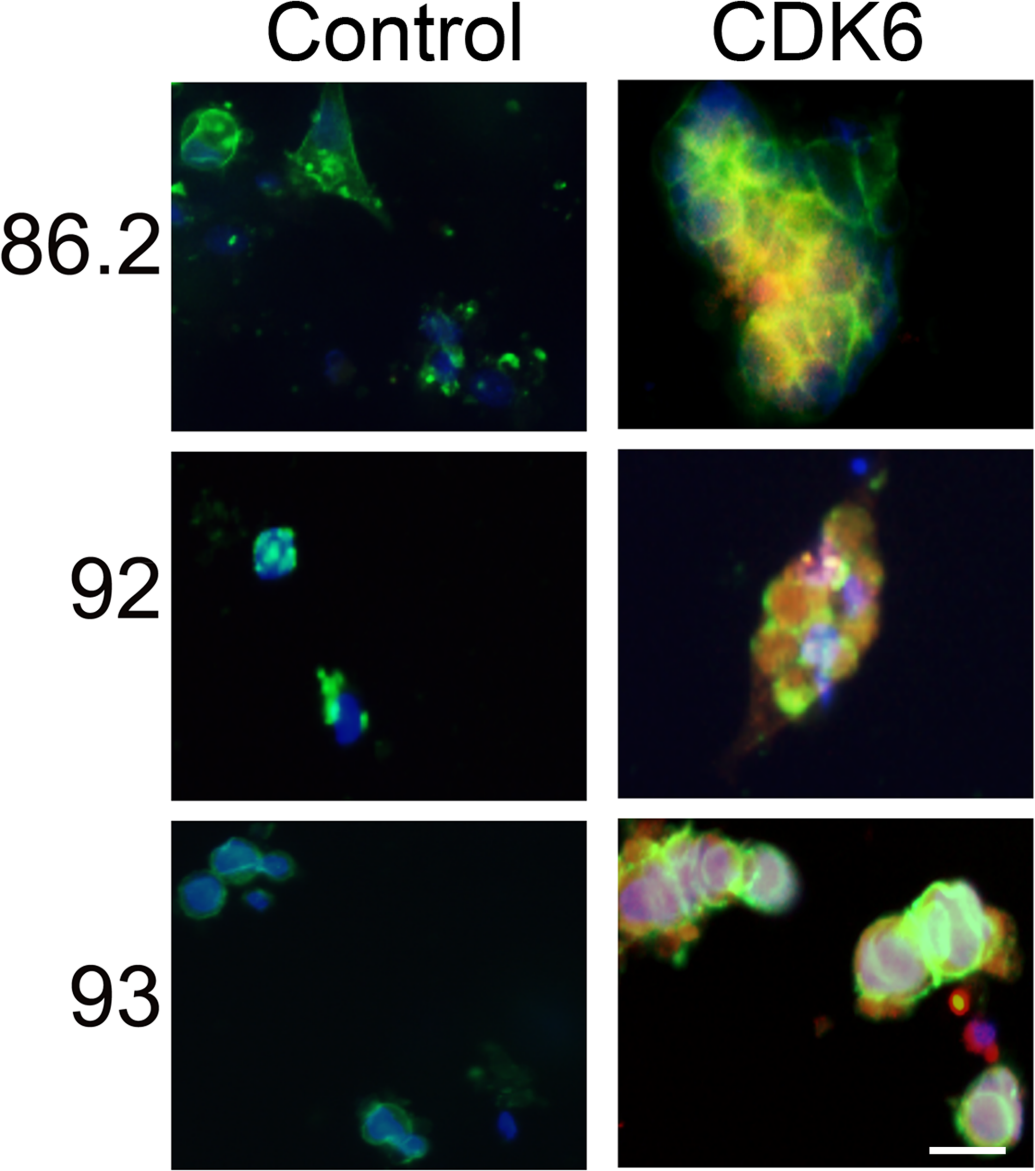
CDK6 overexpression induced proliferation of LuCaP PDX cells *in vitro*. LuCaP 86.2, 92 and 93 cells were lentivirally transduced to overexpress CDK6 and cultured *in vitro* to assess proliferation. Positive Ki67 indicated that CDK6 overexpression facilitated proliferation in these cells. Green, EpCAM; Red, Ki67; Blue, DAPI. Magnification: 200x. Scale bar: 20 μm.

To validate upregulation of CDK6 promoted LuCaP PDX cell proliferation, we infected LuCaP 86.2, 92, and 93 cells with lentivirus containing constitutively active CDK6 vector and examined the proliferation. LuCaP 86.2, 92, and 93 cells normally did not proliferate in the absence of BMSC, however ectopic expression of CDK6 promoted cell proliferation as evidenced by the positive Ki67 staining (Fig 5).

## Discussion

PCa cells may remain quiescent/dormant for years and proliferate to form active metastases at distant sites. Little is known to date about the mechanisms underlying the induction and release from dormancy in PCa cells that reside in the bone marrow. Major reasons include the lack of patient specimens and relevant human *in vitro* and *in vivo* models. We previously reported a potential dormancy signature associated with DTC isolated from PCa patients with no evidence of disease [30]. Here, instead of using immortalized PCa cell lines that readily proliferate *in vitro*, we used clinically relevant PDX cells to examine the mechanism underlying direct cell-cell interaction to restore cell proliferation. We characterized three LuCaP PCa PDXs residing on the human bone marrow stroma, which displayed quiescent/dormant and proliferating phenotypes depending on the cell seeding density. Our results showed that tumor cell-cell contact induced cell proliferation which may represent dormancy escape via activation of β_1_ integrin associated with universal downregulation of TGFB2 signaling and upregulation of MLCK activation/CDK6 in PCa PDXs.

Recent studies in other solid tumors, such as in the head, neck, and breast, have implicated elements of the cytoskeletal migration and adhesion machinery in the activation of indolent tumor cells [4–7, 38–41]. β_1_ Integrin is critical for the initiation of tumorigenesis and the maintenance of the proliferative capacity of tumors [33, 41]. In PCa, we found that cell-cell contact, both between tumor cells and with an underlying stroma, was associated with the activation of β_1_ integrin and was essential to facilitate the growth of quiescent PCa xenograft cells *in vitro*. While cell-cell contact has to our knowledge not been directly reported as a requirement for dormancy release, several mechanisms associated with the migration and adhesion of tumor cells have been implicated in this process. Specifically, activation of α_5_β_1_ integrin has been shown to release human squamous carcinoma and breast cancer cells from dormancy [6, 7, 39, 41], and activation of α_5_ β_1_ integrin induce cell adhesion and migration [42, 43] as well as proliferation on extracellular matrix [44]. Thus, it is not surprising that given an opportunity to come into contact within the bone marrow microenvironment, these normally quiescent LuCaP PDX cells begin to proliferate *in vitro*.

Activation of β_1_ integrin has been shown to induce the downregulation of TGF β2 and Cdc42 [45, 46]. Global gene expression analysis on the reactivated cells versus dormant cells highlighted a decrease in TGF β2 signaling in proliferating PCa PDX cells which was consistent with the observation that TGF β2 induced dormancy of malignant DTC in head and neck squamous cell carcinoma [47]. This is in concordance with the data from clinically derived DTC that *TGFB2* expression is higher in PCa patients with no evidence of disease when compared to patients with advanced disease [35]. Furthermore, Cdc42 was implicated in activation of p38 and growth arrest in cancers [7] and was downregulated in proliferating PCa PDX cells in the current study. Of note, MLCK is a well-known downstream effector of adhesion- and motility-mediated mechanisms including β_1_ integrin [9, 10, 38, 40], and its activation has been linked to cell survival [40]. In our study, the downregulation of CDC42 and TGFB2 pointed to a possible activation of MLCK leading to cell proliferation. Indeed, introduction of constitutively active MLCK alone was adequate to induce proliferation in LuCaP PDX cells that normally retain dormant *in vitro*. Conversely, C4-2B cells, which readily proliferate *in vitro*, were growth suppressed upon treatment with the MLCK inhibition ML-7. These data correlated well with a previous study by Barkan and colleagues that demonstrated quiescence *in vitro* and inhibition of metastatic outgrowth of various breast cancer cell lines upon inhibition of MLCK [38].

**Fig 6:**
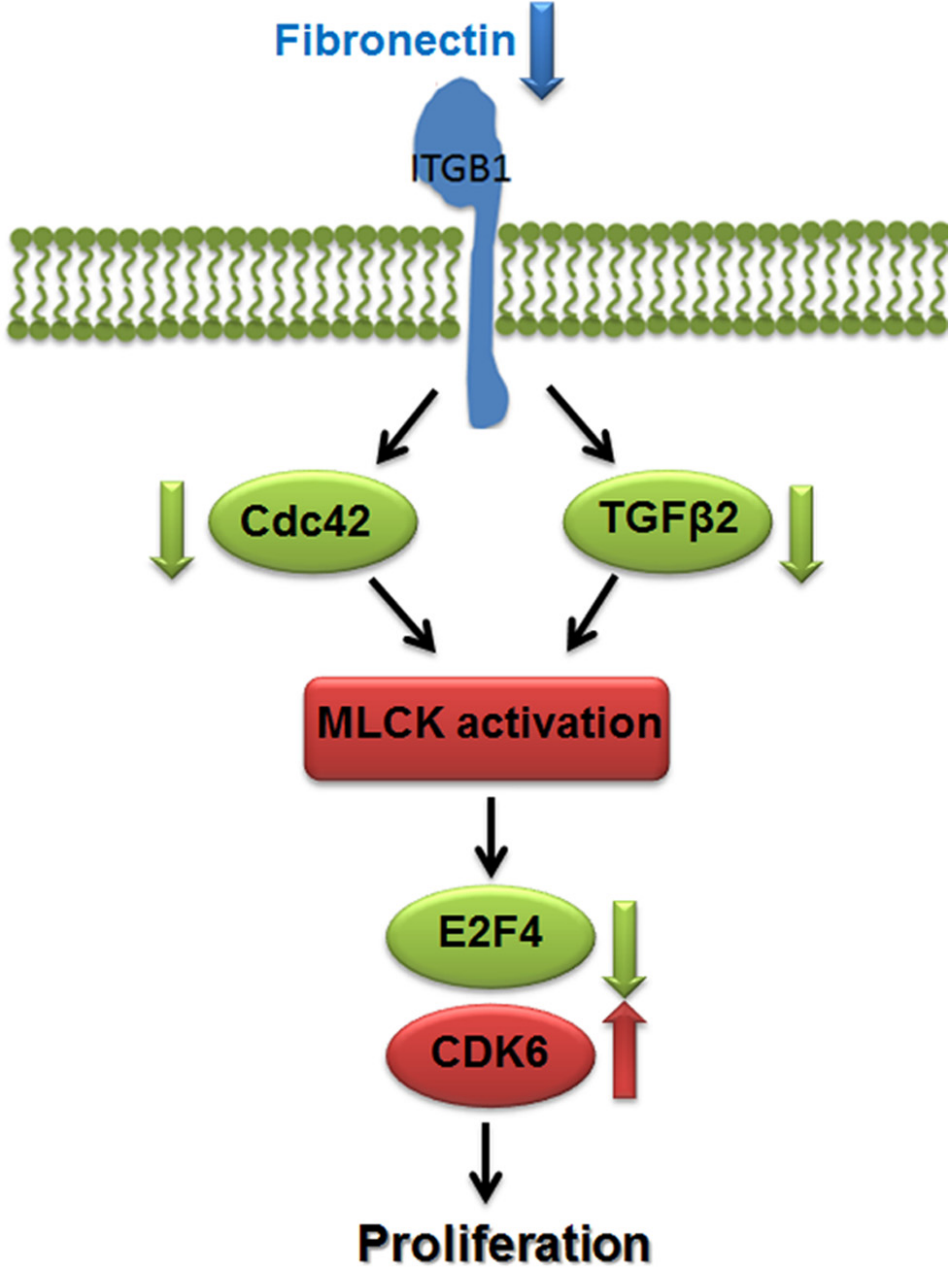
Potential mechanism for PCa release from quiescence/dormancy. Decreased fibronectin activation on β_**1**_ integrin downregulates TGF β2 and Cdc42 resulting in activation of MLCK. This activity leads to the deactivation of growth suppressor like E2F4 and activation of cell cycle regulator CDK6, promoting cell proliferation.

In the current study, activation of MLCK resulted in an upregulation of CDK6 in all three LuCaP PDX lines. CDK 6 associates with cyclin D1 to transition cells through the G1 phase of the cell cycle [48] and is regulated by the androgen receptor (AR) [49]. Instead of acting as a primary regulator, AR may act as an enhancer for CDK6 expression because *CDK6* upregulation upon MLCK activation was 5–7 fold higher in the AR-positive LuCaP 92 and LuCaP 86.2 cells compared to the upregulation in AR-negative neuroendocrine LuCaP 93 cells [26, 27]. On the other hand, E2F4 is known to act in conjunction with Smad3 as a cofactor for TGF transcription [50], which has been shown to induce apoptosis in PCa [51, 52]. Furthermore, E2F4 has been demonstrated to enforce G2 arrest in C4-2B cells in response to genotoxic stress [53]. We observed a downregulation of the E2F4 in proliferating LuCaP PDXs expressing activated MLCK, suggesting that the downregulation in E2F4 may be allowing LuCaP PDX cells to progress through the cell cycle and escape quiescence/dormancy (Fig 6).

Collectively, we presented the first *in vitro* model demonstrating non-proliferating PCa PDX cells resumed proliferation on human bone marrow stromal microenvironment. These models provide evidence to support that direct cell-cell interaction promotes cell proliferation partly via β_1_ integrin activation. A clinically interesting but not yet addressed question is whether or not a patient showing an increased number of DTC (i.e. increased chance of DTC contact with each other) will result in an increased rate of tumor cell proliferation and hence metastatic outgrowth. Maintaining disseminated PCa cells in a dormant, indolent state is an attractive clinical prospect as is inducing active PCa cells to become dormant. However, such treatments require an intimate understanding of PCa dormancy mechanisms. While confirmatory *in vivo* studies are required to conclusively determine a dormancy release mechanism in PCa, these findings represent an important and encouraging first step in the identification of such a mechanism for this heterogeneous disease.

## Supporting Information

S1 Fig C4-2B cells grow on a monolayer of bone marrow stromal cells (BMSC) when seeded sparsely. A) C4-2B cells were seeded sparsely (50 cells/cm^2^) on BMSC, cells were fixed with ice-cold methanol and fluorescently stained for Ki67 to assess proliferation. Green, EpCAM; Red, Ki67, Blue, DAPI. Magnification: 200x. Scale bar: 50 μm. B) EpCAM-positive cells were counted on day 1 and day 7. Data are presented as mean±S.D of two independent experiments. **p<0.01 when compared to day 1.
(PDF)

S2 Fig Cell cycle analysis of ML-7 treatment on C4-2B cells. A) A representative histogram of DAPI-stained C4-2B cells treated with either DMSO or ML-7 (10μM) for 24h or 48h. Cell cycle was analyzed by flow cytometry. B) Percentage of cells in G1, S, and G2/M phase of C4-2B cells treated with DMSO or ML-7. Data are presented as mean±S.D of two independent experiments. *p<0.05 when compared to the DMSO control.
(PDF)
