# Supplementary material for: Cellular Adhesion Promotes Prostate Cancer Cells Escape from Dormancy
Source: PLoS One. 2015 Jun 19;10(6):e0130565. doi: 10.1371/journal.pone.0130565 (PMC4475050; doi:10.1371/journal.pone.0130565)
Supplement: S1 Fig — A) C4-2B cells were seeded sparsely (50 cells/cm2) on BMSC, cells were fixed with ice-cold methanol and fluorescently stained for Ki67 to assess proliferation. Green, EpCAM; Red, Ki67, Blue, DAPI. Magnification: 200x. Scale bar: 50 μm. B) EpCAM-positive cells were counted on day 1 and day 7. Data are presented as mean±S.D of two independent experiments. **p<0.01 when compared to day 1. (PDF) [file pone.0130565.s001.pdf]

Supplementary Figure 1

A

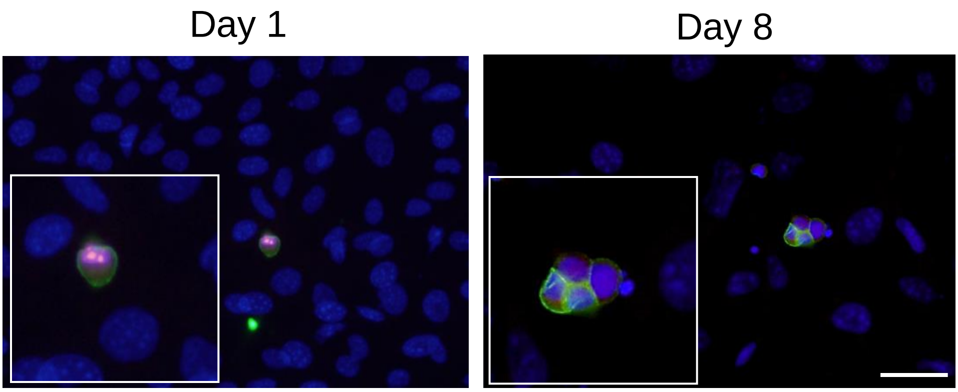

B

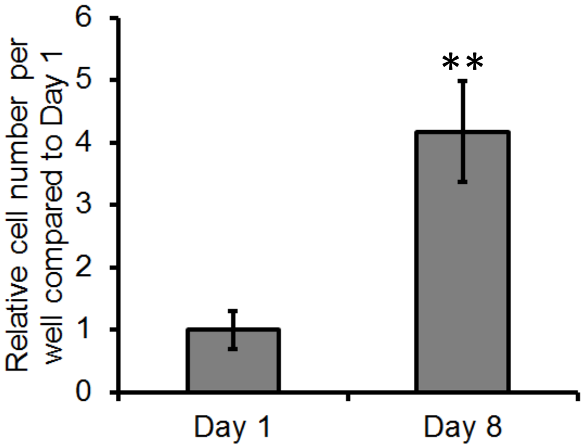

S1 Fig. C4-2B cells grow on a monolayer of bone marrow stromal cells (BMSC) when seeded sparsely. A) C4-2B cells were seeded sparsely (50 cells/cm^2) on BMSC, cells were fixed with ice-cold methanol and fluorescently stained for Ki67 to assess proliferation. Green, EpCAM; Red, Ki67, Blue, DAPI. Magnification: 200x. Scale bar: 50 µm. B) EpCAM-positive cells were counted on day 1 and day 7. Data are presented as mean±S.D of two independent experiments. **p<0.01 when compared to day 1.
