# Supplementary material for: Cellular Adhesion Promotes Prostate Cancer Cells Escape from Dormancy
Source: PLoS One. 2015 Jun 19;10(6):e0130565. doi: 10.1371/journal.pone.0130565 (PMC4475050; doi:10.1371/journal.pone.0130565)
Supplement: S2 Fig — A) A representative histogram of DAPI-stained C4-2B cells treated with either DMSO or ML-7 (10μM) for 24h or 48h. Cell cycle was analyzed by flow cytometry. B) Percentage of cells in G1, S, and G2/M phase of C4-2B cells treated with DMSO or ML-7. Data are presented as mean±S.D of two independent experiments. *p<0.05 when compared to the DMSO control. (PDF) [file pone.0130565.s002.pdf]

Supplementary Figure 2

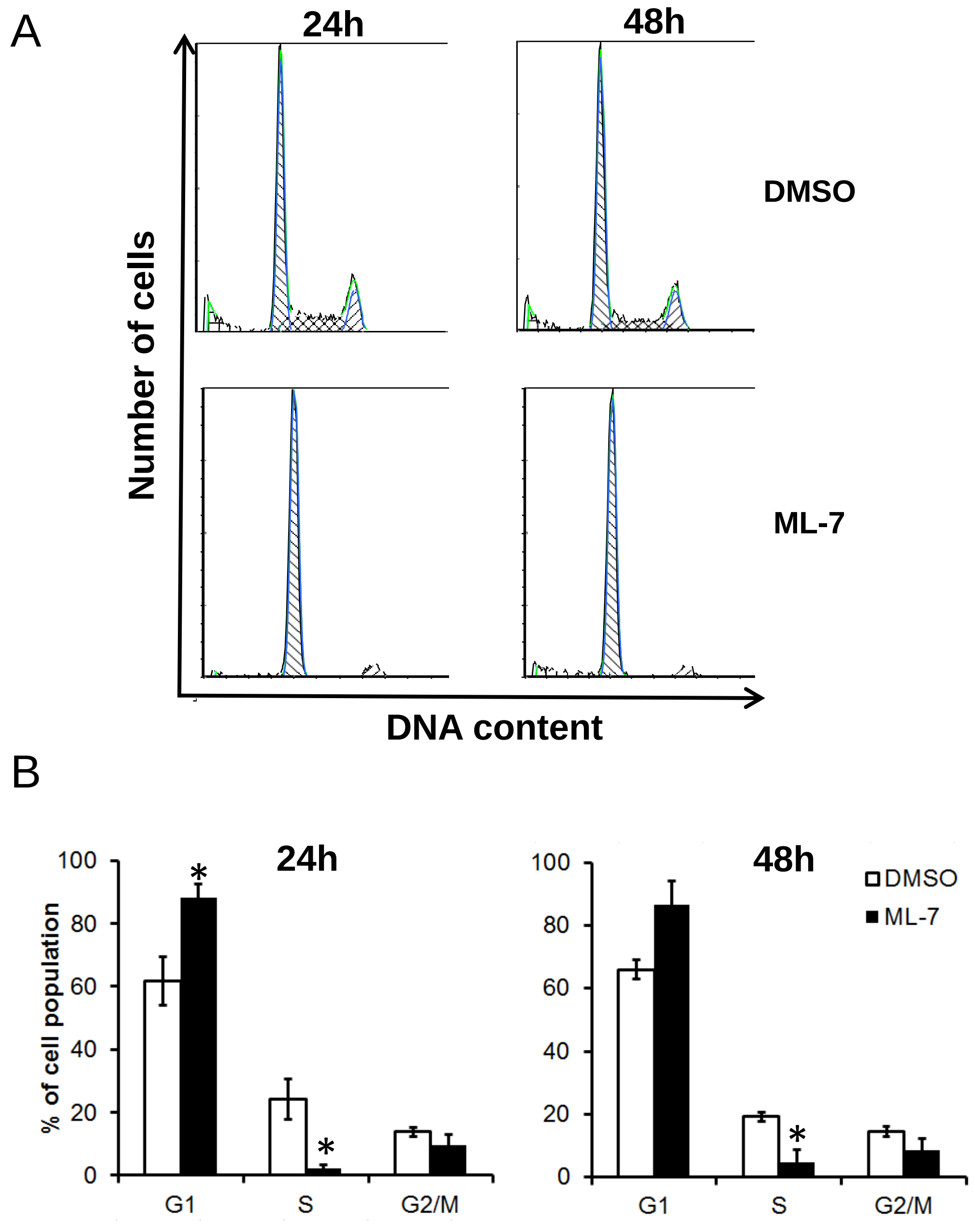

S2 Fig. Cell cycle analysis of ML-7 treatment on C4-2B cells. A) A representative histogram of DAPI-stained C4-2B cells treated with either DMSO or ML-7 (10μM) for 24h or 48h. Cell cycle was analyzed by flow cytometry. B) Percentage of cells in G1, S, and G2/M phase of C4-2B cells treated with DMSO or ML-7. Data are presented as mean±S.D of two independent experiments. *$p<0.05$ when compared to the DMSO control.
